# A retrospective study on *Escherichia coli* bacteremia in immunocompromised patients: Microbiological features, clinical characteristics, and risk factors for shock and death

**DOI:** 10.1002/jcla.23319

**Published:** 2020-04-08

**Authors:** Xiaoyan Tao, Haichen Wang, Changhang Min, Ting Yu, Yi Luo, Jun Li, Yongmei Hu, Qun Yan, Wen' en Liu, Mingxiang Zou

**Affiliations:** ^1^ Department of Clinical Laboratory Xiangya Hospital Central South University Changsha China; ^2^ Faculty of Laboratory Medicine Xiangya School of Medicine Central South University Changsha China

**Keywords:** bacteremia, *Escherichia coli*, outcome, phylogenetic analysis, virulence

## Abstract

**Background:**

To evaluate clinical features, bacterial characteristics, and risk factors for shock and mortality of immunocompromised patients with *Escherichia coli* bacteremia.

**Methods:**

A nearly 6‐year retrospective study of *E coli* bacteremia in 188 immunocompromised patients at Xiangya Hospital was conducted. Demographic, clinical, and laboratory data were documented. Phylogenetic background and virulence factors of *E coli* isolates were detected by polymerase chain reaction. Risk factors for shock and mortality were also investigated.

**Results:**

Of all 188 *E coli* isolates, most prevalent virulence factors were *fimH* (91.0%), followed by *traT* (68.6%) and *iutA* (67.0%), while *papG allele I*, *gafD,* and *cdtB* were not detected. Phylogenetic group D was dominant (42.0%) among all isolates, and group B2 accounted for 17.6%, while group A and B1 accounted for 28.2% and 12.2%, respectively. In univariate analysis, *ibeA* and *cnf1* were associated with mortality, which were not found in multivariate regression analysis. 22.3% of patients suffered shock, and 30‐day mortality rate was 21.3%. MDR (HR 2.956; 95% CI, 1.091‐8.012) was the only risk factor for shock, while adult (HR 0.239; 95% CI, 0.108‐0.527) was a protective factor. Multivariate analysis revealed that shock (HR 4.268; 95% CI, 2.208‐8.248; *P* < .001) and Charlson index > 2 (HR 2.073; 95% CI, 1.087‐3.952; *P* = .027) were associated with fatal outcome.

**Conclusions:**

*Escherichia coli* bacteremia was highly lethal in immunocompromised patients, and host‐related factors played major roles in poor prognosis, while bacterial determinants had little effect on outcome. This study also provided additional information about the virulence and phylogenetic group characteristics of *E coli* bacteremia.

## 1 INTRODUCTION

The gram‐negative bacilli *Escherichia coli* strains form part of the normal microflora of the gastrointestinal tract, but are also common isolates of gram‐negative bloodstream infection (BSI) worldwide.^1^ In addition, *E coli* is an important cause of both community‐onset and hospital‐acquired bacteremia.^2^, ^3^, ^4^ *Escherichia coli* is the leading cause of BSIs, with an incidence ranging from 31.9% to 81.0% among major gram‐negative species in 28 European countries.^5^ Moreover, the increasing incident of multidrug resistant (MDR) *E coli* strains is concerning. For example, in different region of China the isolation rate of ESBL *E coli* in BSI is significantly increased to more than 50.0%.^6^, ^7^ MDR strains are closely associated with appreciable mortality and carried with great economic loss.^8^, ^9^, ^10^ Thus, there is an urgent need to understand factors associated with the causes and outcomes of these infections, in an attempt to reduce their incidence and severity and initiate appropriate therapies.

Virulence factors (VF), for example, hemolysins, adhesins, iron‐acquisition systems, cytotoxins, and siderophores, play an important role in the infection.^11^, ^12^ They are essential for the interaction between *E coli* and its host, to overcome host defenses, invade host tissues, and trigger a local inflammatory response. There have been a number of studies describing the epidemiology, risk factor, and outcome of *E coli* BSIs in different countries.^6^, ^13^ Several VFs, such as *afa*, *iroN,* and *cvaC*, were reported to be associated with mortality.^14^

Immunocompromised patients are more likely to develop bloodstream infection.^15^ And these patients, usually suffering from immunosuppression, chemotherapy, particularly steroids, stem cell transplant, and long‐term hospitalization simultaneously, have relatively poor prognosis and high mortality rate.^16^ Thus, early treatment of bacteremia is clinically important to ultimate clinical outcome. Yet, information about virulence genes and phylogenetic groups of *E coli* strains isolated from immunocompromised patients in mainland China remains scarce and incomplete. The objective of this retrospective study was to assess the clinical features and outcome of *E coli* BSIs in immunocompromised patients and to study the molecular epidemiology of *E coli* isolates.

## 2 MATERIALS AND METHODS

### 2.1 Patients and study design

This retrospective study was conducted at Xiangya Hospital Central South University, a 3,500‐bed teaching hospital in Changsha, Hunan Province, China, from March 2013 to December 2018. Immunocompromised patients diagnosed with at least one blood culture positive for *E coli* bacteremia were included in the study. Outpatients and patients without complete medical records were excluded from the study. Clinical data were collected through a retrospective review of the electronic medical records, including demographic characteristic, clinical and microbiological data, underlying disease, laboratory data at the time of bacteremia onset, results of antimicrobial susceptibility testing, 30‐day mortality, and other relevant information.

The study was approved by the Ethics Committee of Xiangya Hospital, Central South University. No informed consent was taken because this study did not cause additional medical procedure.

### 2.2 Definition

Bloodstream infection was defined as the isolation of organisms from at least one bottle of blood culture specimens from patients with compatible clinical signs or symptoms. The date of the first positive blood culture (index culture) was regarded as the date of bacteremia onset. If the first culture had been drawn more than 48 hours after admission to the hospital, the infection was classified as hospital‐onset; otherwise, it was classified as community‐acquired. Neutropenia was defined as an absolute neutrophil count (ANC) of <500 cells/mm^3^.^17^ Immunocompromised status was defined as any patients with at least one of the following factors: active malignancy or cancer receiving chemotherapy, history of stem cell transplantation or solid‐organ transplantation on immunosuppressive agents, immunosuppressive therapy (including steroid therapy of prednisone ≥10 mg/d or its equivalent administered for ≥7 days), or other underlying immune deficiency. Polymicrobial infection was defined as the presence of microorganisms other than *E coli* identified from blood samples regardless of whether the isolates came from the same or different blood culture sets during the bacteremia period. The isolates that were resistant to three or more categories were defined as multidrug resistant (MDR).^18^ The isolates were regarded as carbapenem‐resistant Enterobacteriaceae (CRE) if they were resistant to any carbapenems by in vitro antibiotic sensitivity test. Prior antibiotic treatment was defined as the receipt of any systemic antibiotic >48 hours in the preceding 30 days.^19^ Appropriate empirical antimicrobial therapy was defined that at least one active antimicrobial agent effective to the organism by in vitro test was administered within 48 hours after the onset of bacteremia. The impact of comorbidities was determined by the Charlson comorbidity index (CCI).^20^

### 2.3 Antimicrobial susceptibility testing

Blood cultures (BD BACTECTM Peds Plus Culture Vial, Becton Dickinson) containing 8‐10 mL blood from adults or 1‐3 mL from children were incubated with an automated system (BACTECTM FX 200, Becton Dickinson). Identification of microorganisms was performed with a MALDI‐TOF MS (Bruker Daltonik GmbH), and antibiotic susceptibility tests were conducted with a VITEK^®^ system (bioMérieux), except for cefoperazone‐sulbactam, which is determined by Kirby‐Bauer disk diffusion method. The Clinical and Laboratory Standards Institute (CLSI) criteria were used to define the susceptibility of the antibiotics, and the susceptibility of cefoperazone‐sulbactam was referred to cefoperazone.

### 2.4 Detection of virulence genes

The detection of virulence genes was done by multiplex PCR. Boiling method was used to extract DNA. Based on Johnson and Stell,^21^ the reactions were slightly adjusted and divided into five pools as follows: in pool 1: PAI, *papA*, *fimH*, *ibeA,* and *papEF*; in pool 2: *fyuA*, *sfa/focDE*, *iutA*, *papG allele III,* and *bmaE*; in pool 3: *hlyA*, *papG I*, *kpsMT II*, *nfaE*, *papC,* and *focG*; in pool 4: *traT*, *papG allele II*, *cvaC*, *cdtB,* and *gafD*; and in pool 5: *afa/draBC*, *cnf1*, *papG allele I*, *papG allele II* and *III*, *sfa,* and K5. PCR products were electrophoresis in 1.0% agarose gel. The gels were visualized using Gel Doc TM XR image analysis station (Bio‐Red).

### 2.5 Phylogenetic analysis

A multiplex PCR method was utilized to detect chuA, yjaA gene, and a DNA fragment TspE4.C2. The PCR steps were as follows: denaturation for 4 minutes at 94°C, 30 cycles of 5 seconds at 94°C and 10 seconds at 59°C, and a final extension step of 5 minutes at 72°C. The amplification products were separated in 1% agarose gels. After electrophoresis, the gel was photographed under UV light, and the strains were assigned to the phylogenetic groups (group A, B1, B2, or D) by use of a dichotomous decision tree.^22^

### 2.6 Statistical analysis

For continuous variables, results were expressed as mean standard deviation (*SD*) or median with interquartile range (IQR) and categorical variables using percentages of the group from which they were derived. To evaluate continuous variables, Student's *t* test (for normally distributed variables) and the Mann‐Whitney *U* test (for variables that did not have normal distribution) were used. Categorical variables were analyzed by the chi‐squared or Fisher's exact test when appropriate. *P* values < .05 were considered statistically significant. All variables that were associated with shock and death in the univariate analysis (*P* < .05) were entered into a multivariate logistic regression analysis. All statistical analyses were carried out using SPSS version 22.0.

## 3 RESULTS

### 3.1 Demographic and clinical characteristics

A total of 188 patients were identified during the study period. The median age was 34.9 years old (range 1‐73), and 94 were female (50.0%). Most patients had underlying disorders, in which hematological diseases were the most prevalent, including acute myeloid leukemia (35.1%), acute lymphoblastic leukemia (24.5%), and other hematological diseases (24.5%). Moreover, 21 patients suffered from solid tumors (11.2%) and 9 patients had autoimmune diseases (4.8%). Among the total of 188 episodes, the most common comorbidity was liver damage (11.7%), followed by diabetes (7.4%) and hypertension (3.7%). According to the clinical and laboratory data, 114 (60.6%) patients had primary sepsis, 39 (20.7%) patients had bacteremia from a gastrointestinal origin, 26 (13.8%) had respiratory origin, 6 (3.2%) had urinary origin, and 3 (6.3%) cases of bacteremia had other origins.

In our study, all patients received empirical antibiotics, among which 172 were appropriate (91.5%), and a majority of patients (129, 68.6%) had a history of antibiotics within 30 days before the onset of bacteremia. Of the 188 patients with *E coli* BSIs, 149 developed neutropenia (79.3%) with an average duration of 15.1 ± 10.1 days and 84 were unrecovered (44.7%). A majority of *E coli* bacteremia was classified as hospital‐onset infections (158, 84%), while others were community‐acquired. Polymicrobial infection was noticed in 29 patients (15.4%): 23 cases were infected with another bacterium, and 5 cases were infected by other two bacteria, while one was infected by another three. *Klebsiella pneumoniae* was found in 10 episodes, followed by fungi in six, *Acinetobacter baumannii* in four, *Enterococcus faecium* and *Pseudomonas aeruginosa* each in three, and several other bacteria. In the study, 42 (22.3%) patients suffered shock and the rate of 30‐day mortality was 21.3% (Table 1).

**TABLE 1:** Demographic and clinical characteristics of 188 episodes of *Escherichia coli* bacteremia

Characteristics	All patients
	n = 188, n (%)
Sex, female	94 (50.0)
Age (y), median (range)	34.9 (1‐73)
Underlying disorders	
Acute myeloid leukemia	66 (35.1)
Acute lymphoblastic leukemia	46 (24.5)
Solid tumor	21 (11.2)
Autoimmune diseases	9 (4.8)
Others	46 (24.5)
Comorbidities	
Liver disease	22 (11.7)
Diabetes mellitus	14 (7.4)
Hypertension	7 (3.7)
Adult	137 (72.9)
Appropriate empirical therapy	172 (91.5)
Neutropenia	
Neutropenia	149 (79.5)
Length of days, mean ± SD	15.4 ± 10.1
Unrecovered neutropenia	84 (44.7)
MDR	128 (68.1)
CRE	12 (6.4)
Charlson comorbidity index	2.96 ± 1.68
Polymicrobial infection	29 (15.4)
Antibiotics exposure, 30 d	129 (68.6)
RDW	15.44 ± 3.08
Source	
Primary	114 (60.6)
Urinary tract	6 (3.2)
Respiratory	26 (13.8)
Intra‐abdominal	39 (20.7)
Others	3 (1.6)
Infections at admission	
Community acquired	30 (16.0)
Hospital associated	158 (84.0)
Shock	42 (22.3)
30‐d mortality	40 (21.3)

Abbreviations: CRE, carbapenem‐resistant Enterobacteriaceae; MDR, multidrug resistant; RDW, red blood cell distribution width.

### 3.2 Antibiotic susceptibility

Among all *E coli* strains, the MDR rate was 68.1% and 12 isolates, accounting for 6.4%, were identified as CRE, as shown in Table 2. The *E coli* isolates exhibited a high susceptibility to imipenem (93.6%), amikacin (96.3%), piperacillin‐tazobactam (86.7%), and cefoperazone‐sulbactam (86.7%). The resistance rates for other antibiotics tested were more than 35%, of which ciprofloxacin was the highest (69.1%). Compared to non‐MDR *E coli*, the antibiotic resistance rate of the MDR group was generally higher, except for amikacin (94.5% v 100%, *P* = .099), which showed high sensitivity to both MDR and non‐MDR *E coli* strains (Table 2).

**TABLE 2:** Antibiotic resistance rates of the *Escherichia coli* strains (n, %)

Antibiotic	All isolates	MDR	non‐MDR	*P* value
	(n = 188)	(n = 128)	(n = 60)	
Piperacillin‐tazobactam	25 (13.3)	25 (19.5)	0 (0.0)	<.001
Cefoperazone‐sulbactam	25 (13.3)	25 (19.5)	0 (0.0)	<.001
Ceftazidime	69 (36.7)	62 (48.4)	7 (11.7)	<.001
Ceftriaxone	129 (68.6)	110 (85.9)	19 (31.7)	<.001
Cefepime	69 (36.7)	65 (50.8)	4 (6.7)	<.001
Imipenem	12 (6.4)	12 (9.4)	0 (0.0)	<.001
Aztreonam	87 (46.3)	80 (62.5)	7 (11.7)	<.001
Ciprofloxacin	128 (69.1)	116 (90.6)	12 (20.0)	<.001
Levofloxacin	124 (66.0)	113 (88.3)	11 (18.3)	<.001
Gentamycin	95 (50.5)	84 (65.6)	11 (18.3)	<.001
Amikacin	7 (3.7)	7 (5.5)	0 (0.0)	.099
Trimethoprim‐sulfamethoxazole	124 (66.0)	104 (81.3)	20 (33.3)	<.001

Abbreviation: MDR: multidrug resistant.

### 3.3 Distribution of VFs and phylogenetic groups

Regarding the virulent genes, the most prevalent VFs were *fimH* (91.0%), followed by *traT* (68.6%), *iutA* (67.0%), *fyuA* (56.9%), and *kpsMT II* (49.5%), and the less prevalent VFs were *papG allele II* (1.1%), *hlyA* (1.6%), *focG* (1.6%), *bmaE* (2.1%), *nfaE* (3.2%), *afa/draBC* (3.7%), *cvaC* (5.3%), *sfaS* (7.4%), *sfa/focDE* (8.0%), and *ibeA* (8.5%), while *papG allele I*, *gafD,* and *cdtB* were not detected in all isolates.

The most common phylogenetic group is group D with the highest proportion of 42.0% (79/188), followed by group A with 28.2%, group B2 with 17.6%, and group B1 with 12.2% (Table S1).

### 3.4 Risk factors for shock in patients with *Escherichia coli* BSI

Among all patients, 42 patients suffered shock, accounting for 22.3%, as shown in Table 3. In the univariate analysis, compared with non‐shock group, the average age of shock group was younger (24.6 years old vs 37.9 years old, *P* < .001) and there were more patients had a history of antibiotic exposure within 30 days (85.7% vs 63.7%, *P* = .008) in shock group. Nearly all the patients in the shock group had neutropenia, with the exception of one patient, and the difference was significant compared with the non‐shock group (97.6% vs 74%, *P* < .001). Other factors associated with shock were underlying disorders (*P* = .004), MDR (80.5% vs 62.3%, *P* = .015). As for VFs and phylogenetic groups, none of them was associated with shock (Table S2).

**TABLE 3:** Univariate analysis of risk factors for shock and 30‐d mortality of *Escherichia coli* BSIs in immunocompromised patients

Characteristics	Shock	Non‐shock	*P* value	95% CI	Non‐survival	Survival	*P* value	95% CI
	n = 42, n (%)	n = 146, n (%)			n = 40, n (%)	n = 148, n (%)		
Age (y), median (range)	24.6 (2‐67)	37.9 (1‐73)	**<.001**		37 (2‐69)	34.3 (1‐73)	.418	
Sex, female	20 (47.6)	74 (50.7)	.861	0.445‐1.758	16 (40.0)	78 (52.7)	1.000	0.467‐2.352
Underlying disorders			**.004**				.357	
Acute myeloid leukemia	11 (26.2)	55 (37.7)			12 (30.0)	54 (36.5)		
Acute lymphoblastic leukemia	18 (42.9)	28 (19.2)			8 (20.0)	38 (25.7)		
Solid tumor	1 (2.4)	20 (13.7)			4 (10.0)	17 (11.5)		
Autoimmune diseases	0 (0.0)	9 (6.2)			1 (2.5)	8 (5.4)		
Others	12 (28.6)	34 (23.3)			15 (37.5)	31 (20.9)		
Comorbidities								
Liver disease	2 (4.8)	20 (13.7)	.171	0.071‐1.407	4 (10.0)	18 (12.2)	1.000	0.225‐2.521
Diabetes mellitus	1 (2.4)	13 (8.9)	.199	0.032‐1.965	3 (7.5)	11 (7.4)	1.000	0.268‐3.808
Hypertension	1 (2.4)	6 (4.1)	1.000	0.067‐4.865	1 (2.5)	6 (4.1)	1.000	0.071‐5.191
Adult	19 (45.2)	118 (80.8)	**<.001**	0.094‐0.408	29 (72.5)	108 (73.0）	1.000	0.446‐2.137
Appropriate empirical therapy					35 (87.5)	137 (92.6)	.339	0.183‐1.723
Neutropenia								
Neutropenia	41 (97.6)	108 (74.0)	**<.001**	1.918‐108.516	34 (85.0)	115 (77.7)	.384	0.629‐4.206
Length of days, mean ± SD					15.9 ± 12.4	15.3 ± 9.3	.791	
Unrecovered neutropenia					31 (77.5)	53 (35.8)	**<.001**	2.734‐13.943
MDR	35 (80.5)	92 (62.3)	**.015**	1.219‐7.064	29 (72.5)	99 (66.9)	.570	0.602‐2.829
CRE	3 (7.1)	9 (6.2)	.732	0.302‐4.536	5 (12.5)	7 (4.7)	.135	0.832‐9.610
Charlson comorbidity index	2.92 ± 1.66	2.67 ± 1.70	.963		3.45 ± 1.89	2.82 ± 1.60	**.023**	
Polymicrobial infection	6 (14.3)	23 (15.7)	1.000	0.337‐2.356	8 (20.0)	21 (14.2)	.214	0.712‐4.303
Antibiotics exposure within 30 d	36 (85.7)	93 (63.7)	**.008**	1.352‐8.647	34 (85)	95 (64.2)	**.012**	1.247‐8.018
RDW	15.80 ± 4.39	15.34 ± 2.60	.475		15.87 ± 3.83	15.33 ± 2.85	.650	
Source			.722				.145	
Primary	27 (64.3)	87 (59.6)			21 (52.5)	93 (62.8)		
Urinary tract	0 (0.0)	6 (4.1)			0 (0.0)	6 (4.1)		
Respiratory	5 (11.9)	21 (14.4)			10 (25.0)	16 (10.8)		
Intra‐abdominal	10 (23.8)	29 (19.9)			9 (22.5)	30 (20.3)		
Others	0 (0.0)	3 (2.1)			0 (0.0)	3 (2.0)		
Infections at admission			.483	0.547‐4.276			.467	0.584‐3.513
Community acquired	5 (11.9)	25 (17.1)			8 (20.0)	22 (14.9)		
Hospital associated	37 (88.1)	121 (82.9)			32 (80.0)	126 (85.1)		
Shock					19 (47.5)	23 (15.5)	**<.001**	2.292‐10.551

Abbreviations: CRE, carbapenem‐resistant Enterobacteriaceae; MDR, multidrug resistant; RDW, red blood cell distribution width.

In the multivariate logistic analysis, MDR (HR 2.956; 95% CI, 1.091‐8.012) was the only risk factor of shock, while adult (HR 0.239; 95% CI, 0.108‐0.527) was the protective factor (Table 4).

**TABLE 4:** Multivariate analysis of risk factors for shock of *Escherichia coli* BSIs in immunocompromised patients

Variables	HR	*P* value	95% CI
Adult	0.239	<.001	0.108‐0.527
MDR	2.956	.033	1.091‐8.012

Abbreviations: HR, hazard ratio; MDR, multidrug resistant.

### 3.5 Risk factors for mortality in patients with *Escherichia coli* BSI

The characteristic of survivors and non‐survivors is shown in Table 3. Univariate analysis showed that the mortality of *E coli* BSIs was associated with higher CCI score (3.45 ± 1.89 vs 2.82 ± 1.6; *P* = .023), presence of shock (47.5% vs 15.5%, *P* < .001), and unrecovered neutropenia (77.5% vs 35.8%, *P* < .001). 129 of patients were treated with antibiotics 30 days prior admission, and the history of antibiotic treatment showed a significant difference between survival and non‐survival (85.0% vs 64.2%, *P* = .012). Meanwhile, there were no significant differences in mortality among *E coli* BSI patients that received appropriate empirical treatment compared with inappropriate empirical treatment (87.5% vs 92.6%, *P* = .339).

**FIGURE 1:**
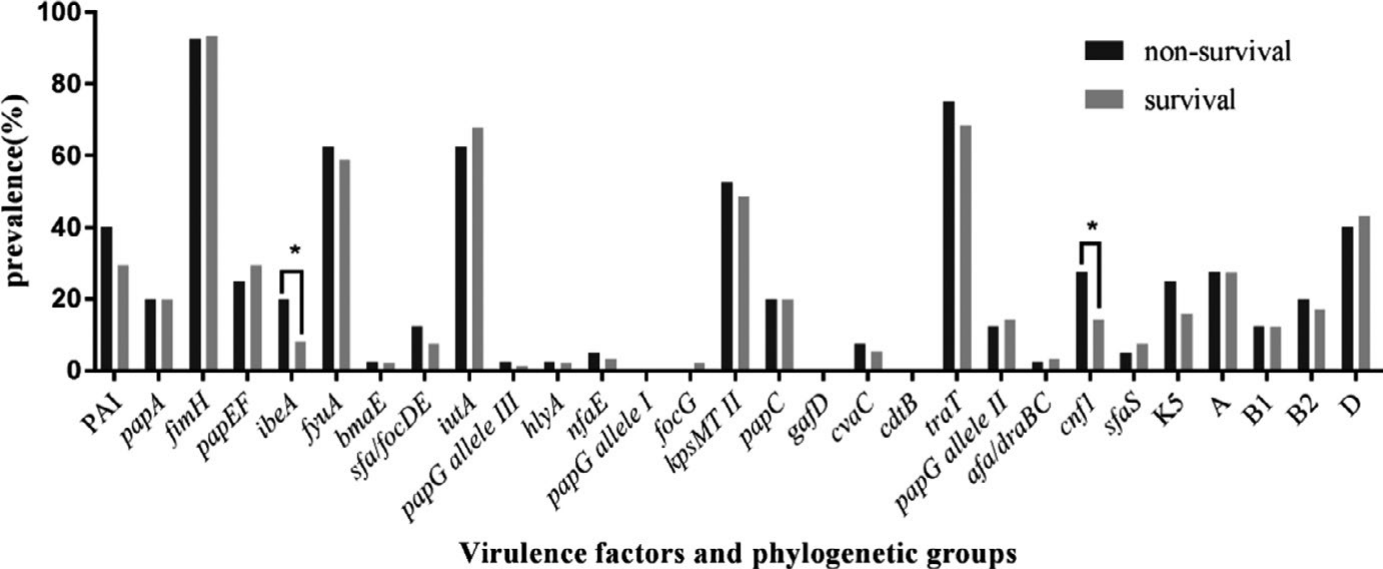
Differences in the prevalence of virulence factors and phylogroups among non‐survival and survival groups (for detailed results see Table S1). Note: statistically significant results with *P < .05

The two virulence genes *ibeA* (20% vs 5.4%, *P* = .007) and *cnf1* (27.5% vs 11.5%, *P* = .015) were positively correlated with mortality. However, these correlations were not found in multivariate analysis. In our study, there was no significant association between phylogenetic group distribution and mortality (Figure 1).

Multivariate analysis revealed that Charlson comorbidity index > 2 (HR 2.073, 95% CI, 1.087‐3.952; *P* = .027) and shock (HR 4.268, 95% CI, 2.208‐8.248; *P* < .001) were positively associated with mortality (Table 5).

**TABLE 5:** Multivariate analysis of risk factors for mortality in immunocompromised patients with *Escherichia coli* BSIs

Variables	HR	*P* value	95% CI
Charlson comorbidity index > 2	2.073	.027	1.086‐3.952
Shock	4.268	<.001	2.208‐8.248

Abbreviation: HR: hazard ratio.

## 4 DISCUSSION

Bloodstream infection is an important cause of death in immunocompromised patients. In recent years, *E coli* has gradually become the most common pathogen of bloodstream infection and received extensive attention because of its severe antibiotic resistance. In this study, we analyzed the demographic data, microbiological characteristics, and outcomes of immunocompromised patients with *E coli* BSIs to identify the risk factors of shock and mortality.

During the study period, a total of 188 *E coli* strains were isolated. Among the 25 VFs detected, *fimH* (type 1 fimbria), *iutA* (aerobactin), *fyuA* (yersiniabactin siderophores), and *traT* (conjugal transfer surface exclusion protein) were the most prevalent, which were also reported high prevalence in *E coli* extraintestinal infections in many other studies including bacteremia.^21^, ^23^, ^24^ The *fimH* gene mediates bacterial attachment and invasion to bladder epithelial cells and promotes biofilm formation. Thus, *fimH* plays an important role in urinary tract infection.^25^ The *iutA* and *fyuA* genes, present in 67% and 56.9% of the isolates, respectively, are included in iron uptake systems. The presence of *fyuA* gene was associated with increased mortality reported by Mora‐Rillo et al.^26^ The serum resistance‐related virulence factor *traT*, detected in 68.6% of isolates, can subvert complement‐mediated killing and is also essential for bacteremia.^21^ The high prevalence of these different kinds of VFs all contributed to occurrence of *E coli* BSIs.

In the univariate analysis, *ibeA* and *cnf1* were associated with 30‐day mortality. The virulence gene *cnf1* is a cytotoxic necrotizing factors, encoding a protein that affects cellular function, including inflammation and inhibition of neutrophil phagocytosis and chemotactic activities of neutrophils.^27^ The prevalence rates reported in other studies were relatively low, ranging from 7.2% to 24%.^26^, ^28^, ^29^ *ibeA*, present in 8.5% of isolates, was associated with central nervous system infections, particularly in newborns, and has been shown to be involved in interacting with surface proteins of the brain microvascular endothelial cells and facilitating invasion of the central nervous system.^30^ Previous study also reported 10% prevalence of *ibeA* and was correlated with increased mortality.^31^ In another study from China, it reported that siderophores *iroN* and *iss* were associated with 30‐day mortality in univariate analysis.^32^

In current study, the 25 VFs detected did not show any differences between shock and non‐shock groups in multivariate analysis, and the same results were also found in survival and non‐survival groups. It suggested that host determinants may override bacterial VFs in determining the mortality of *E coli* BSIs. Further researches are needed to explore the influence of VFs on the mortality of *E coli* BSIs.

Regarding to the distribution of phylogenetic groups, we found that group D was the most prevalent group, with 79 isolates accounting for 42.0%, while the prevalence of group B2 was only 17.6%, which is different from the results of most previous studies. Typically, *E coli* strains isolated from bacteremia mainly belong to group B2, followed by group D, and they had been confirmed as highly virulent strains.^33^ One study observes that B2 group had a prevalence of 52.6%, while group D only accounted for 18.4%.^34^ Few studies shared similar results with this study, except for a research by Bozcal et al,^28^ who found group D dominated by a rate of 38.14% in *E coli* strains isolated from bacteremia. Up to 40.4% of BSIs were caused by less virulent *E coli* strains, group A and B1, probably due to the immune deficiency state of the host. Like other studies, no impact of phylogenetic distribution on the shock and mortality was demonstrated in this study.

In our study cohort, the rate of shock was 22.3%, which is a little higher than that reported in studies of adult patients with cancer or hematological diseases.^35^, ^36^ However, a previous study reported a rate of 51.1% for shock in pediatric hematological patients with *E coli* BSIs.^37^ In present study, we found that adult was a protective factor for shock, which may also proved that children were more vulnerable to shock. However, because the number of patients included in this study is relatively small, so these results require further studies with larger size in different regions to verify.

Multivariate analysis revealed that MDR was the only risk factor for shock. MDR strains, accounting for 68.1%, showed a high resistance rate to almost all antibiotics except for carbapenem and amikacin, a phenomenon which was in line with the results in other studies.^38^, ^39^ Considering that large proportion of patients in this cohort received antibiotics treatment 30 days before the acquisition of BSIs (68.6%) and the high rate of hospital‐associated BSIs (84.0%), the MDR rate is within expectation. Studies have reported an association between MDR and inadequate empirical antibiotic therapy.^40^ In previous studies, the correlation between drug resistance and poor prognosis has been reported.^26^, ^41^ In our study, MDR, although being an important factor for shock, showed no difference between survivals and non‐survivals. Other study showed the same results.^19^ Part of the reasons for this result may be that the vast majority of patients in this study cohort received appropriate empirical antibacterial therapy.

In this study, the mortality rate cohort was 21.3%, similar to the mortality rates reported by other researchers from 10.3% to 33.3%.^14^, ^26^, ^42^ However, considering the high rate of appropriate empirical treatment, 91.5% for the whole cohort, the mortality is still disturbing. A relatively high proportion of hospital‐associated infection (84.0%) was observed, compared to other studies. In a 10‐year multicenter study by Scheuerman et al,^43^ only 41.3% of hospital‐associated infection rates were reported in adults patients with *E coli* BSIs. In addition to shock, CCI scores are also a reliable factor in predicting mortality, which has been well elaborated in both immunocompetent and immunocompromised patients.^35^, ^43^, ^44^ As for antibiotic exposure within 30 days, the antibiotic exposure rate in the non‐survival group was higher than that in the survival group (34/40, 85.0% vs 95/148, 64.2%, *P* = .012). Patients with cancer or hematological diseases usually receive periodic chemotherapy, which often leads to neutropenia, and are more susceptible to infection. Empirical use of antibiotics, a vital means of preventing infection during neutropenia, is recommended by the American Infectious Diseases Association.^45^ Therefore, high frequency of antibiotic treatment is sometimes inevitable for immunocompromised patients. However, it is still necessary for clinicians to strictly control the application of antibiotics in order to improve survival of patient and reduce the incidence of antibiotic resistance.

In summary, this study evaluated the host and bacterial factors affecting the prognosis of *E coli* BSIs. We found that host‐related factors played a major role in the *E coli* BSIs, while VFs and phylogenetic groups of *E coli* showed little effect on prognosis of immunocompromised patients. Our study also showed that MDR rate of *E coli* was still concerning and was the only risk factor related to shock, while adult was the protective factor. The study revealed that shock and Charlson's comorbidities index >2 were two independent factors for mortality of *E coli* BSIs.

There are several limitations in our study. First, our data were based on the local epidemiology of a single center and cannot be generalized. Further studies of a larger number of patients from different regions are necessary to assess the influence of these determinants on the outcome of *E coli* BSIs. Moreover, due to the retrospective nature of this study, several patients were excluded from the study cohort because of incomplete clinical data, which may result in a slight degree of selection bias. At last, the present study failed to associate any particular bacterial determinant with the risk of shock and death. Thus, further research should be conducted on the genetic features of these pathogens, such as pathogenicity islands and plasmids associated with the virulence.

## CONFLICT OF INTEREST

No conflict of interests is declared.

## Supporting information

Table S1‐S2
