# Supplementary material for: A retrospective study on Escherichia coli bacteremia in immunocompromised patients: Microbiological features, clinical characteristics, and risk factors for shock and death
Source: J Clin Lab Anal. 2020 Apr 8;34(8):e23319. doi: 10.1002/jcla.23319 (PMC7439330; doi:10.1002/jcla.23319)
Supplement: Supplementary file 1 — Table S1‐S2 [file JCLA-34-e23319-s001.docx]

**Table S1**. Distribution of virulence genes and phylogenetic groups and the relationship with 30-day mortality in 188 *E. coli* BSIs

|  | **all patients** | **non-survival** | **survival** | ***P* value** | **95% CI** |
| --- | --- | --- | --- | --- | --- |
|  | **(n=188)** | **(n=40)** | **(n=148)** |  |  |
| **Virulence genes** | |  |  |  |  |
| *PAI* | 56(29.8) | 16(40.0) | 40(27.0) | 0.122 | 0.868-3.732 |
| *papA* | 36(19.1) | 8(20.0) | 28(18.9) | 1.000 | 0.446-2.576 |
| *fimH* | 171(91.0) | 37(92.5) | 134(90.5) | 1.000 | 0.352-4.723 |
| *papEF* | 52(27.7) | 10(25.0) | 42(28.4) | 0.698 | 0.378-1.872 |
| *ibeA* | 16(8.5) | 8(20.0) | 8(5.4) | **0.007** | 1.527-12.534 |
| *fyuA* | 107(56.9) | 25(62.5) | 82(55.4) | 0.475 | 0.655-2.749 |
| *bmaE* | 4(2.1) | 1(2.5) | 3(2.0) | 1.000 | 0.125-12.246 |
| *sfa/focDE* | 15(8.0) | 5(12.5) | 10(6.8) | 0.320 | 0.633-6.138 |
| *iutA* | 126(67.0) | 25(62.5) | 101(68.2) | 0.570 | 0.375-1.606 |
| *papG allele III* | 2(1.1) | 1(2.5) | 1(0.7) | 0.381 | 0.231-61.625 |
| *hlyA* | 3(1.6) | 1(2.5) | 2(1.4) | 0.514 | 0.165-21.183 |
| *nfaE* | 6(3.2) | 2(5.0) | 4(2.7) | 0.609 | 0.334-10.737 |
| *papG allele I* | 0 | 0 | 0 | / | / |
| *focG* | 3(1.6) | 0(0.0) | 3(2.0) | 1.000 | / |
| *kpsMTII* | 93(49.5) | 21(52.5) | 72(48.6) | 0.723 | 0.580-2.348 |
| *papC* | 36(19.1) | 8(20.0) | 28(18.9) | 1.000 | 0.446-2.576 |
| *gafD* | 0 | 0 | 0 | / | / |
| *cvaC* | 10(5.3) | 3(7.5) | 7(4.7) | 0.446 | 0.403-6.624 |
| *cdtB* | 0 | 0 | 0 | / | / |
| *traT* | 129(68.6) | 30(75.0) | 99(66.9) | 0.346 | 0.627-3.283 |
| *papG allele II* | 25(13.3) | 5(12.5) | 20(13.5) | 1.000 | 0.320-2.610 |
| *afa/draBC* | 7(3.7) | 1(2.5) | 6(4.1) | 1.000 | 0.071-5.191 |
| *cnf1* | 28(14.9) | 11(27.5) | 17(11.5) | **0.015** | 1.239-6.897 |
| *sfaS* | 14(7.4) | 2(5.0) | 12(8.1) | 0.738 | 0.128-2.781 |
| K5 | 31(16.5) | 10(25.0) | 21(14.2) | 0.147 | 0.860-4.724 |
| **Phylogenetic groups** | |  |  | 0.956 |  |
| A | 53(28.2) | 11(27.5) | 42(28.4) |  |  |
| B1 | 23(12.2) | 5(12.5) | 18(12.2) |  |  |
| B2 | 33(17.6) | 8(20.0) | 25(16.9) |  |  |
| D | 79(42.0) | 16(40.0) | 63(42.6) |  |  |

**Table S2**. The relationship of virulence genes and phylogenetic groups with shock in 188 *E. coli* BSIs

|  | **shock** | **non-shock** | ***P* value** | **95% CI** |
| --- | --- | --- | --- | --- |
|  | **n=42** | **n=146** |  |  |
| **Virulence genes** |  |  |  |  |
| *PAI* | 13(31.0) | 43(29.5) | 1.000 | 0.510-2.261 |
| *papA* | 7(16.7) | 29(19.9) | 0.668 | 0.326-2.000 |
| *fimH* | 35(83.3) | 136(93.2) | 0.066 | 0.131-1.035 |
| *papEF* | 9(21.4) | 43(29.5) | 0.336 | 0.288-1.481 |
| *ibeA* | 4(9.5) | 12(8.2) | 0.759 | 0.358-3.854 |
| *fyuA* | 21(50.0) | 86(58.9) | 0.377 | 0.350-1.389 |
| *bmaE* | 1(2.4) | 3(2.1) | 1.000 | 0.118-11.477 |
| *sfa/focDE* | 4(9.5) | 11(7.5) | 0.747 | 0.389-4.288 |
| *iutA* | 27(64.3) | 99(67.8) | 0.711 | 0.416-1.756 |
| *papG allele III* | 0(0.0) | 2(1.4) | 1.000 | / |
| *hlyA* | 0(0.0) | 3(2.1) | 1.000 | / |
| *nfaE* | 1(2.4) | 5(3.4) | 1.000 | 0.078-6.054 |
| *papG allele I* | 0(0.0) | 0(0.0) | / | / |
| *focG* | 0(0.0) | 3(2.1) | 1.000 | / |
| *kpsMTII* | 22(52.4) | 71(48.6) | 0.728 | 0.585-2.310 |
| *papC* | 7(16.7) | 29(19.9) | 0.668 | 0.326-2.000 |
| *gafD* | 0(0.0) | 0(0.0) | / | / |
| *cvaC* | 2(4.8) | 8(5.5) | 1.000 | 0.176-4.225 |
| *cdtB* | 0(0.0) | 0(0.0) | / | / |
| *traT* | 29(69.0) | 100(68.5) | 1.000 | 0.489-2.154 |
| *papG allele II* | 4(9.5) | 21(14.4) | 0.606 | 0.203-1.938 |
| *afa/draBC* | 2(4.8) | 5(3.4) | 0.654 | 0.264-7.543 |
| *cnf1* | 7(16.7) | 21(14.4) | 0.806 | 0.468-3.029 |
| *sfaS* | 3(7.1) | 11(7.5) | 1.000 | 0.251-3.553 |
| K5 | 8(19.0) | 23(15.8) | 0.639 | 0.517-3.063 |
| **Phylogenetic groups** |  |  | 0.926 |  |
| A | 13(31.0) | 40(27.4) |  |  |
| B1 | 5(11.9) | 18(12.3) |  |  |
| B2 | 8(19.0) | 25(17.1) |  |  |
| D | 16(38.1) | 63(43.2 |  |  |
